# Primary care practice and cancer suspicion during the first three COVID-19 lockdowns in the UK: a qualitative study

**DOI:** 10.3399/BJGP.2021.0719

**Published:** 2022-08-09

**Authors:** Claire Friedemann Smith, Brian D Nicholson, Yasemin Hirst, Susannah Fleming, Clare R Bankhead

**Affiliations:** GP and National Institute for Health and Care Research academic clinical lecturer;; GP and National Institute for Health and Care Research academic clinical lecturer;; Institute of Epidemiology and Health, University College London, London.; Nuffield Department of Primary Care Sciences, University of Oxford, Oxford.; Nuffield Department of Primary Care Sciences, University of Oxford, Oxford.

**Keywords:** cancer, COVID-19, early diagnosis, general practice, remote consulting

## Abstract

**Background:**

The COVID-19 pandemic has profoundly affected UK primary care, and as a result the route to cancer diagnosis for many patients.

**Aim:**

To explore how the pandemic affected primary care practice, in particular cancer suspicion, referral, and diagnosis, and how this experience evolved as the pandemic progressed.

**Design and setting:**

Seventeen qualitative interviews were carried out remotely with primary care staff.

**Method:**

Staff from practices in England that expressed an interest in trialling an electronic safety-netting tool were invited to participate. Remote, semi-structured interviews were conducted from September 2020 to March 2021. Data analysis followed a thematic analysis and mind-mapping approach.

**Results:**

The first lockdown was described as providing time to make adjustments to allow remote and minimal-contact consultations but caused concerns over undetected cancers. These concerns were realised in summer and autumn 2020 as the participants began to see higher rates of late-stage cancer presentation. During the second and third lockdowns patients seemed more willing to consult. This combined with usual winter pressures, demands of the vaccine programme, and surging levels of COVID-19 meant that the third lockdown was the most difficult. New ways of working were seen as positive when they streamlined services but also unsafe if they prevented GPs from accessing all relevant information and resulted in delayed cancer diagnoses.

**Conclusion:**

The post-pandemic recovery of cancer care is dependent on the recovery of primary care. The COVID-19 pandemic has highlighted and exacerbated vulnerabilities in primary care but has also provided new ways of working that may help the recovery.

## INTRODUCTION

The COVID-19 pandemic, which was officially recognised by the World Health Organization in March 2020,^1^ has had a profound impact on health care in the UK. The UK government managed COVID-19 through a series of lockdowns^2^ that put substantial restrictions on activities of the public and businesses, and mandated a pause to all but essential health care with limited face-to-face contact.^3^^,^^4^ The pandemic initiated significant changes in ways of working as efforts were made to maintain provision of care under these conditions.^5^ The impact on primary care in particular has been substantial as it provides 90% of clinician–patient contacts in the NHS in England through almost 1 million consultations daily.^6^^,^^7^

At the beginning of the pandemic, NHS England instructed all GP practices to adopt a ‘total triage’ model where patient requests were triaged, the majority of consultations took place remotely, and face-to-face appointments were provided to those for whom it was deemed necessary.^8^ In April 2020 the British Medical Association and the Royal College of General Practitioners issued guidance on prioritising clinical activities using a red (should be postponed if prevalence of COVID-19 is high), amber (should continue if time and resources allow), and green (should continue regardless of prevalence of COVID-19) system.^9^ One ‘green’ activity was the assessment of new potential cancers, although it was also recommended that investigations be done remotely if possible and referrals made without seeing the patient.

GPs are central to cancer diagnosis in the NHS. In 2017, 61% of new cancers were detected through referrals made by GPs;^10^ however, only a small proportion of presentations to primary care result in serious disease diagnosis.^11^ Many patients later diagnosed with cancer present with non-specific symptoms making tools such as clinical intuition and safety netting (the practice of monitoring patients with symptoms possibly indicative of an underlying serious illness such as cancer until they are resolved or explained) particularly important.^11^^–^^15^ These essential components of the consultation were markedly disrupted when face-to-face consultations were largely replaced by telephone or online alternatives to combat SARS-CoV-2 transmission.^16^

Although some research has examined cancer diagnosis in primary care during the first lockdown in England,^17^^,^^18^ to the authors’ knowledge, no study has reported on how the situation evolved throughout the following year. In March 2020 the authors launched the CASNET2 study,^19^ shortly before the start of the UK government’s measures were put in place in response to the COVID-19 pandemic. The purpose of the study was to trial a new electronic health records-compatible safety-netting tool in general practice. In light of the increased pressures, it was decided that the CASNET2 study would be paused to allow practices to focus on their COVID-19 response. The authors took this opportunity to speak to staff in CASNET2 practices about their experiences during the pandemic. Staff from practices randomised to trial the tool as well as staff from practices who had expressed an interest were contacted and asked if they would like to take part in an interview study. The aim of this study was to discuss the experience of NHS primary care staff throughout the three lockdowns in the UK and explore how the detection of cancer functioned during the pandemic.

**Table:** How this fits in

The COVID-19 pandemic caused huge disruption to primary care services and as the majority of cancers are first detected in primary care these changes have had an impact on the normal routes to cancer diagnosis. In this study, participants described how the initial decrease in patient consultations allowed them to reconfigure their practices, but also resulted in higher rates of late cancer presentations and diagnosis after the first lockdown. During the second and third lockdowns, participants described how they used all means available to them to get patients who had already experienced delays investigated. The greater use of technology during the consultation was thought to help with streamlining some processes, but participants were also cautious about how easily vital information could be omitted and prevent the GP from seeing the full picture of the patient’s condition or make the use of clinical intuition difficult. These results illustrate how the balance was tipped in favour of reducing the risk of SARS-CoV-2 during the COVID-19 pandemic to the detriment of the assessment of cancer risk.

## METHOD

### Recruitment

Staff from practices participating in the CASNET2 study were contacted. These practices were all located in England. Both randomised and non-randomised practices were invited as pressure on primary care was high and so a larger number of practices would help with the recruitment of a sufficient sample for this study. Staff were told that the interviews were to explore their experiences during the COVID-19 pandemic, particularly in relation to how patients presented, were investigated or safety netted, and followed up when they had symptoms that could indicate cancer. Any staff member involved in the care of patients or the organisation of the practice could participate. All staff who responded to the invitation were interviewed.

### Patient and public involvement

CASNET2 has a patient and public involvement panel that has met regularly throughout the project. Before the interviews began, the panel met to discuss the project priorities and the areas of safety netting and cancer care that were of interest to patients. This was considered when writing the interview schedule but the patient and public involvement group did not have direct input into it. Since the completion of the interviews, the panel has met to discuss the findings and highlight important results from the patient perspective.

### Interviews

**Figure 1.**
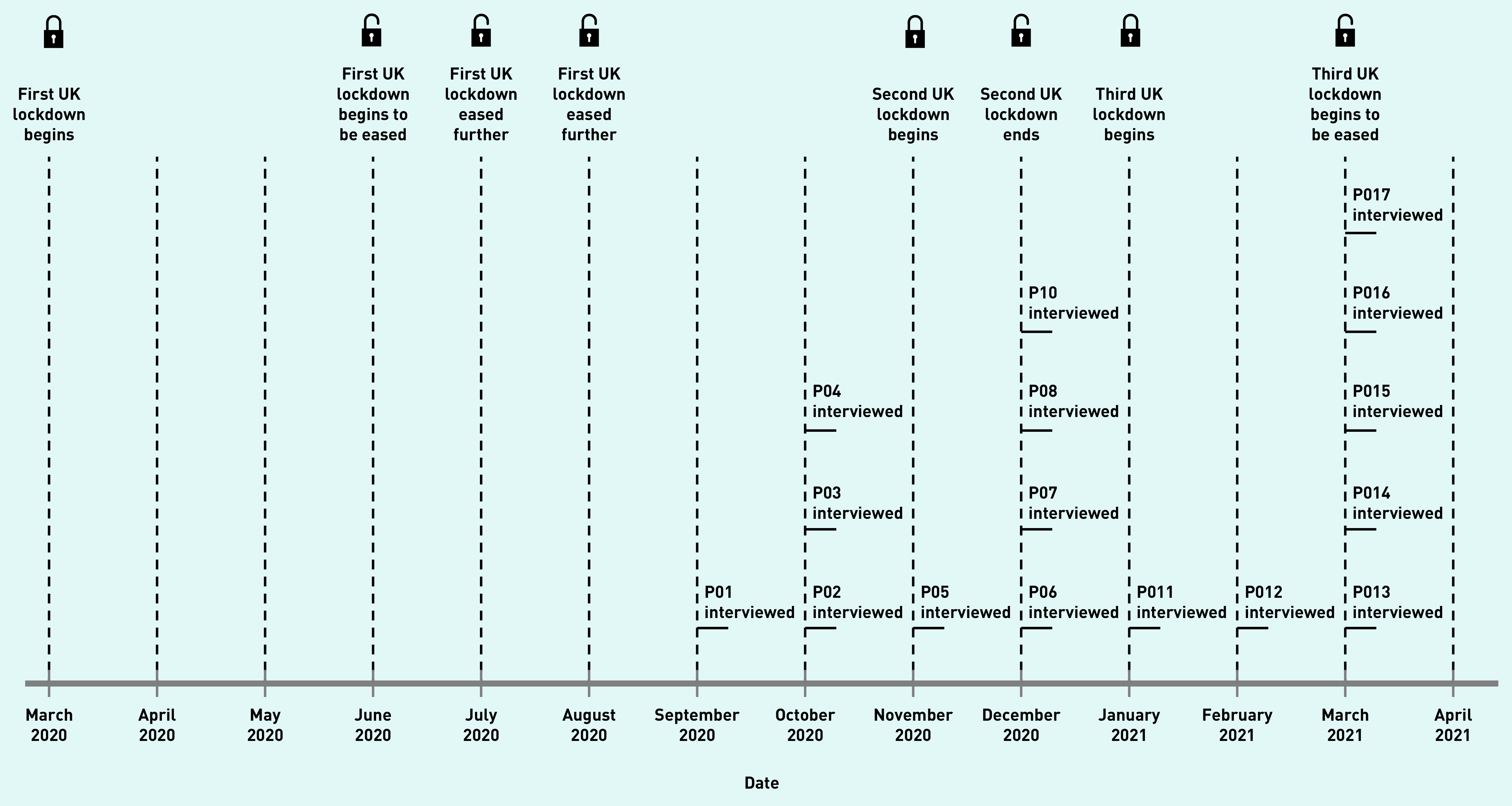
*UK lockdowns and study timeline. Lockdown dates as reported by the Institute for Government Analysis.^23^*

Semi-structured interviews lasting an average of 43 minutes (range 30–57) were conducted by the first author between September 2020 and March 2021 (Figure 1) either over the telephone (*n* = 2) or over Microsoft Teams (*n* = 15). The first author monitored the interviews for data saturation^20^ and judged that it had been reached at the beginning of March 2021. No new themes arose after interview 12. Any primary care staff scheduled to be interviewed after this were interviewed to gather additional perspectives on the themes. Written informed consent was obtained from the interviewees, and was confirmed at the start of each interview.

Interviews began with a discussion of the interviewee’s career to date, and the size and make-up of their practice, before considering their experience of the COVID-19 pandemic and its impact on cancer care. The interviews also briefly covered tools to facilitate safety netting in primary care. These questions formed the basis of later user experience interviews for the CASNET2 study. The interview schedule is available in Supplementary Appendix S1, where questions relevant to this study are highlighted in yellow.

### Analysis

The interviews were digitally recorded, transcribed verbatim, and transcripts were uploaded into NVivo (version 12). Thematic analysis^21^ was used to encode the data and collate these codes into themes by the first author. Issues arising within the themes and links between the themes were mapped using a mind-mapping ‘one sheet of paper’ (OSOP)^22^ method. A summary of the OSOP findings was presented to the research team and the patient and public involvement group by the first author and discussed. Following discussion, the themes deemed to be of most interest and importance to the patient and public involvement group and that addressed the study aims were reported.

## RESULTS

Interviews were conducted with 17 primary care staff from 17 practices (Box 1). Seven of the interviewees were female and 10 male, and they had been qualified for between 3 years and 30 years.

**Box 1.** Interview participants

**Participant**	**Role in practice**	**Sex**	**Years’ GP experience**	**Geographical region**	**Date of interview**
P01	GP	Male	5	Midlands	September 2020
P02	GP	Male	29	South West	October 2020
P03	GP	Male	28	South West	October 2020
P04	GP	Female	16	North East	October 2020
P05	GP	Male	3	South West	November 2020
P06	GP	Male	12	South East	December 2020
P07	GP	Male	30	Midlands	December 2020
P08	Nurse	Female	22^a^	South West	December 2020
P09	Practice manager	Female	7^b^	Midlands	December 2020
P10	GP	Male	29	East	January 2021
P11	GP	Female	12	South East	January 2021
P12	GP	Female	4	South East	February 2021
P13	GP	Female	8	South West	March 2021
P14	GP	Female	4	North West	March 2021
P15	GP	Male	12	North East	March 2021
P16	GP	Male	5	North West	March 2021
P17	GP	Male	5	North West	March 2021

a *Years since qualifying as a nurse.*

b *Years with current practice.*

### First lockdown

#### Impact on primary care generally

In general, the participants characterised the first lockdown as *‘a very very uncertain time’* (Participant [P]12, female [F], February 2021) of rapid change when being flexible was essential. The long quotation from P12 in Box 2 is representative of the experiences of participants in this study who described reconfiguring their practice, including the carpark and outdoor areas, the creation of ‘hot’ and ‘cold’ teams, and the introduction of enhanced hygiene procedures.

**Box 2.** Changes to primary care in Lockdown 1

*‘So, we shifted to remote and we also had to put so many things in place,* [reduced the] *number of chairs in the whole waiting room to have the social distancing you know. And we also put lots of marks on the floor, we put lots of cordoning on the floors, even in the meeting rooms and the kitchen, and the outside in the parking lot we blocked off some car parking* [spaces] *.* *‘And then we put in place lots of hand sanitising stations and the receptionists, you know at intervals, they keep cleaning the chairs, wiping everything down. The doors, the chairs, and therefore every clinician, if you do see a face to face you need to wipe out after that patient. Clean the chairs, clean all your equipment, clean the doors, you know, you doff, don, and doff your PPE.* *‘The main thing I would say we also implemented was the isolation centre, which I would say we call it the hot zone. So, we demarcated a part of the surgery and we cordoned it off with all the plastic wraps and things and it was the isolation room. And we also created, luckily, we already had that, another door on that side, so it was separate from the main entrance. And then the special parking lot, so patients would drive, park, and then we also made a walkway, so the walkway went from that door right to the parking, where the patient parked. So immediately as the patient comes down, they just enter that walkway, comes into the isolation room* […] *Then we created a new shower room as well. So, for every clinician that was going in, you just come out, you shower, you don, doff, shower, and then you come out*. *So those were new things that, it took quite a bit of getting used to* [and] *the duty doctors were allocated, they were running shifts to run* [the practice] *.* *‘Then as well there was the CCG* [clinical commissioning group] *allocated certain GPs as hot sites, so, so that you know the mix of suspicion of COVID patients, would not mix with the cold cases, it was called hot and cold cases. They were called hot sites, so these were suspected COVID cases that didn’t have to go to hospital but needed to be seen. And then we also had the cold sites for you know the people that were not suspected of COVID.’* (P12, female, February 2021)

Participants said that during this initial phase of the pandemic there was a lack of strategies from administrative bodies that led to anxiety among practice staff, even *‘blind panic’* (P16, male [M], March 2021) and confusion:

*‘*[at the beginning of lockdown 1] *I think the PCNs* [primary care networks] *were still working out a strategy for face to face, because the, even the practice was waiting on PPE* [personal protective equipment] *and all those kind of things, to make it safer. But* [with] *video and telephone consultations, GPs were still working.’*

(P01, M, September 2020)

The messaging directed at the public, however, was seen as highly effective at deterring patients from contacting general practice. Participants described an initial dramatic reduction in the volume of patients requesting consultations. During this time, practices took the opportunity to prepare and get *‘geared up for remote working’* (P07, M, December 2020).

The uncertainty experienced during this time also extended to patient care. Uncertainty was experienced regarding whether and when patients could be followed up and what would happen once the patient was referred. These uncertainties had implications when cancer was suspected and are discussed in the next theme.

#### Impact on cancer diagnosis

During the first lockdown, participants described how the infrequency with which they saw patients meant that when and how they could follow up a patient was uncertain if there was no immediate reason for the patient to be referred. This added difficulty to managing patients with non-specific symptoms, and required staff to balance the risk of cancer with the risk that either the patient or the staff member caring for them could catch COVID-19:

*‘It kind of felt like you were releasing them into the wild and might not be able to follow them up for a long time.’*

(P05, M, November 2020)

*‘It’s weighing up that risk–benefit to the patient* […] *how worried am I about this patient? Can it wait a little while?* […] *is it worth the risk for this patient, and the staff,* […] *if that phlebotomist went and caught COVID from that patient, could I justify doing that blood test?’*

(P04, F, October 2020)

Under these circumstances, participants described how they might agree some next steps with the patient. These steps ranged from requesting updates or actively following up patients more frequently, to signposting, and could be facilitated by the new digital tools available to GPs and patients:

*‘It’s so easy for them to keep me posted with the symptom now* [referring to eConsult] […] *you have a lot more tools at your fingertips.’*

(P05, M, November 2020)

With the decrease in patients presenting to primary care during the first lockdown, participants described a drop in patients with suspected cancer, which some perceived may have resulted in more advanced cancers presenting during autumn 2020:

*‘So last week in 2 days I saw three late presentations of melanoma* […] *they had been left from March, cos they’d first noticed them in March, but hadn’t come in because of the COVID.’*

(P03, M, October 2020)

Additionally, when patients did consult their GP, increased waiting times to access secondary care investigations resulted in suspected cancer not being investigated for extended periods:

*‘It’s made the diagnostics a little bit harder, sometimes to either access or knowing when you’ve referred, certainly in the first couple of months* [of the pandemic] *, knowing that they’re not going to, nothing’s really going to happen.’*

(P02, M, October 2020)

### Second and third lockdown

#### Impact on primary care generally

Participants described a gradual change *‘back to how things were’* (P01, M, September 2020) following the first lockdown where face-to-face consultations were reintroduced for pre-booked appointments. There was a sense of trepidation from participants interviewed in autumn 2020 that the coming winter would be the hardest part of the pandemic:

*‘April, May, June was a piece of cake, that wasn’t an issue. Our issue I always felt was going to be this September, October, November, and leading up to Christmas, cos for all the obvious reasons, it was going to get busier […] just waiting for that, and everybody uses the term tsunami, but just that massive wave to hit us.’*

(P02, M, October 2020)

Participants also described a change in attitudes where, perhaps out of becoming accustomed to the pandemic or frustrated by having to delay consultation for their medical concerns, patients became much more demanding of consultations with their GP:

*‘Patient attitude changed probably after about the first 2 months, to actually “I want everything sorting, even my cataract, I don’t care about COVID”, and I, I would say that’s stayed.’*

(P04, F, October 2020)

The sense that workload was increasing remained into the autumn and winter of 2020/2021. Participants stated that the second lockdown in November 2020 had little or no impact on the numbers of patients presenting, and additional activities to *‘catch up’* (P10, M, January 2021) kept the workload high. The third lockdown was in force in England from the end of December 2020 to March 2021 and was described as the worst part of the pandemic. During this period a slight drop in routine presentations was described but was offset by much higher cases of COVID-19, staff administering vaccines, and a perception that chronic and mental illnesses were reaching crisis point:

*‘That sort of 6 weeks after January was as bad as anything I’ve ever seen* […] *everything that was kind of prophesised it happened* […] *and was everything kind of that we feared it was going to be.’*

(P16, M, March 2021)

Some participants mentioned the increasingly stressful working environment in primary care, which some believed would decrease staff retention:

*‘I think in contrast to where we were in April last year where people were clapping for health professionals and care workers, it does feel like there is more aggro in comparison to that.’*

(P16, M, March 2021)

#### The impact on cancer diagnosis

Despite the return to more normal patterns of consulting, many of the participants said that they believed the pandemic’s full impact on cancer diagnoses had not been seen yet and were anticipating that the impact would be very bad for patient outcomes:

*‘There’s bound to be some delay in diagnoses, there’s bound to be some though we, we all know that’s going to come and kick us up the backside at some stage, but we don’t know when.’*

(P06, M, December 2020)

Some participants spoke about increases in the proportion of patients they referred to 2-week-wait pathways (urgent referral pathways where the patient should be seen by a hospital specialist for cancer investigations within 2 weeks). They explained that this was often because delayed presentations meant that the time for watchful waiting had passed or patients’ symptoms had developed to the point where they met referral criteria. Participants reported that their thresholds for referral also lowered, partly because of a reduction in direct-access testing meaning that 2-week-wait referral was the only route to investigation for these patients:

*‘There are just more people coming with, with histories that fit a 2-week criteria* […] *and you know the 2-week is the only access really that we have to, for example CT scans* […] *So, I have a lower threshold now I think than I did before because before we had direct access scanning.’*

(P11, F, January 2021)

*‘In GP* [general practice] *we use time as a kind of tool,* [but] *when somebody’s come to you with like 6 months of symptoms, you can’t really use that anymore.’*

(P14, F, March 2021)

There was a mix of opinion on how secondary care had coped as the pandemic progressed. A minority of participants said their local hospitals had *‘coped really remarkably well’* (P11, F, January 2021) but most described tightening referral criteria that led to increased referral rejections and backlogs in 2-week-wait investigations that meant that waits routinely exceeded 2 weeks. This was described as being especially true when the specialty was one that could not easily make use of remote consulting:

*‘I think dermatologists were very good, the colorectal gastro weren’t very good at all. And they’re still not. A 2-week referral for gastro probably still waiting 4 months. Colorectal 4 or 5 weeks. And that’s now* [January 2021] *, the system didn’t work for bowel cancer or GI* [gastrointestinal] *. ENT’s* [ear, nose, and throat] *not much better either actually.’*

(P10, M, January 2021)

One GP who worked shifts in his local emergency department described how he was seeing the consequences of delayed presentation and investigation as an increase in the number of emergency diagnoses of cancer:

*‘I think I’ve probably anecdotally seen more* [cancers diagnosed in accident and emergency] *in the last 6 months than I probably would have in the period before for a couple of years.’*

(P16, M, March 2021)

### Remote consulting and cancer suspicion

There was a mix of experiences of the impact of remote consultations on cancer suspicion. For some participants, the absence of a physical examination when dealing with specific symptoms did not substantially change how they would practise as the patient’s history and knowledge of their own body were sufficient grounds to make a referral. Prior knowledge of patients and information gathered during previous visits also supplemented the remote consultation. For others, the lack of physical examination along with reduced continuity of care meant that remote consultations increased the risk that cancer would be missed:

*‘For example patients who had scans cancelled because of the first wave, weren’t brought in for face-to-face examination, had multiple consultations with different doctors, turned out to have for example ovarian cancer, and* […] *it’s just like that, that Swiss cheese effect, there’s just lots of things that unfortunately happened, because of lockdown,* [that] *I think in normal times wouldn’t have happened.’*

(P11, F, January 2021)

Regarding safety netting, some GPs described how they had become more careful with the advice they gave patients, bearing in mind that it may be difficult for patients to contact their GP. Participants also spoke about how the increased use of technology was changing their processes for safety netting and making digital safety-netting tools more convenient than other methods:

*‘I would say I’m using more of the online safety netting* […] *than when I see patients face to face.’*

(P12, F, February 2021)

*‘We’re already thinking what can we do to safety net better, you know, if someone’s on the phone for a problem more than twice how do we flag that up and bring them in?’*

(P17, M, March 2021)

One of the drawbacks of remote consulting was described as the lack of subtle cues that *‘make your antennae twitch’* (P07, M, December 2020) and facilitated cancer suspicion. As such, a number of participants described having to be more careful for fear of missing something that could indicate a serious underlying condition:

*‘We are missing the face-to-face clues, so if someone comes in through the door, and I can tell whether they’re unwell or not the minute I see them, but over the phone you’ve got to go through a lot more cautious stages to get all the information to document they’re OK.’*

(P03, M, October 2020)

Although a number of participants described benefits to patients being able to use their phone to send images of skin lesions, a greater number expressed concern at missing the full picture in remote consultations when the only opportunity to see the patient was in a (sometimes poor quality) photograph or on a screen. P17 gave two examples of patients where missing detail delayed a cancer diagnosis:

*‘We’ve had a few, well a couple of patients* […] *who had a, something in her axilla, a rash, we thought it was an infected cyst, it certainly looked like that on the photographs* […] *she had a couple of courses of antibiotics and then, so we thought we’re going to have to see her and so she came in* […] *she had a full fungating breast cancer that we hadn’t seen in the photograph* […] *and one of the other doctors mentioned someone who had an ear infection, that we just treated over the phone, when he actually came in, he had a skin cancer on his scalp* […] *and so you know phone and camera is not, it’s not safe.’*

(P17, M, March 2021)

## DISCUSSION

### Summary

During the COVID-19 pandemic, a balancing act was necessitated between reducing the risk of SARS-CoV-2 transmission and appropriately assessing the risk of undetected cancer. During the first UK lockdown, general practice used the quiet period to rapidly reconfigure services to provide remote and minimal contact consulting. The participants in this study reported that this quiet period caused delays to cancer diagnoses as patients refrained from consulting, which they began to see in the summer and autumn of 2020. During the second and third lockdowns, as numbers of patients consulting returned to normal, the participants described how they used all means available to have patients who had already experienced delays investigated. Participants described lowering their risk threshold for referral, using 2-week-wait pathways to access investigations, and modifying their diagnostic approach. Information gathered in pre-pandemic face-to-face encounters facilitated clinical intuition but assessing risk digitally and using ‘gut feeling’ nevertheless remained difficult.

### Strengths and limitations

The strength of this study is that it was possible to gather experiences of the pandemic that spanned the three UK lockdowns and the winter period of 2020/2021, and so this study has been able to report on how practice staff saw the situation evolve and how concerns raised early on in data collection played out. Another strength is that the sample was drawn from across England with geographic spread, including participants based in both rural and urban practices, and in areas with varying prevalence of COVID-19.

The participants were, however, all based in England and so these findings may not reflect the experience of staff working in the other UK nations, especially as the devolved governments did not always follow the same timeline for lockdowns.^23^ A second limitation is that, although the study presents how the views of GPs on consulting and cancer suspicion evolved over the pandemic, follow-up with the initial participants was not undertaken, which would have given a more direct account of changing experiences.

All interviews and coding of data were carried out by one researcher, which may be seen as a limitation as the preconceptions of the researcher may influence how data is coded and interpreted.^24^ To mitigate this, synthesis and interpretation of themes were discussed extensively and agreed by the research team and with the patient and public involvement group. Readers are encouraged to bear in mind the reflexivity statement included at the end of this article when considering the findings of this study.

Finally, this study does not include patients’ perspectives on the changes in primary care or the challenge of having non-COVID-19 symptoms investigated. It is known from recently published research that the public were reluctant to present to primary care even when experiencing red-flag cancer symptoms during and shortly after the first UK lockdown,^25^ but there is still a need for research to explore how attitudes changed as the pandemic continued.

### Comparison with existing literature

The wholesale reconfiguration of primary care during the first lockdown, creation of ‘hot’ and ‘cold’ zones, practices, and teams, repurposing areas for activities such as decontamination, and the spacing out of appointments have been previously reported.^18^^,^^26^ Additional demands from the vaccination programme at a time when COVID-19 was reaching its third peak may have contributed to the increased rates of GP burnout that were mentioned by participants in the current study and elsewhere.^27^ The changes were not all negative though, with both participants in the current study and elsewhere describing how the pandemic facilitated a rapid uptake of technology that normally would have been met with resistance.^28^ The successful uptake of technology, however, cannot be equated to its improving patient outcomes. A number of participants in this study mentioned that technology could hinder the detection of cancer, for example, through poor image quality, and gave examples where not being able to see the wider picture of the patient’s health may be harming outcomes. However, neither the successful uptake nor the suppositions of potential harm are evidence of the impact of these new ways of working on patient outcomes, and this remains to be seen.

Studies have reported reduced rates of consultation for cancer symptoms and of cancer diagnoses during 2020.^17^^,^^29^^–^^32^ Participants in the current study also reported longer waiting times for secondary care investigations and they are not alone in this observation.^17^^,^^33^^,^^34^ Reduced rates of consultation began to disappear following the first lockdown^35^ and participants found that this was accompanied by an influx of patients who had been experiencing symptoms for some time. For these patients, further delay was not felt to be appropriate, and this led to an increased number of referrals later in the study period that was reflected in a report by Cancer Research UK in May 2021.^36^

### Implications for research and practice

The message of stay home to protect the NHS was highly effective during the first part of the pandemic, as evidenced by the period of very few consultations. The intention of this messaging was clearly not to prevent patients with potentially life-threatening conditions from seeking medical help; nevertheless, this was the result.^25^ Balanced messaging and improved public understanding of risk are needed, and how both of these could be achieved should be established. As pandemics are set to become more frequent,^37^ it is important that lessons are learned from COVID-19 to avoid creating a situation where a crisis of non-communicable disease, as may be seen with cancer, follows every pandemic.

Clinicians modified their diagnostic approach as consultation format and consulting behaviour changed throughout the pandemic. The COVID-19 pandemic has indicated some ways of working that could streamline services, for example, through remote consulting and removing the need for physical examination before referral. These modifications could, however, harm the use of other longstanding tools such as clinical intuition. Research is needed to establish the circumstances in which modifications to clinical practice may be effectively used to improve care and whether approaches such as electronic safety-netting tools^19^ may mitigate some of the risk.

This study is one of a growing number that have reported the immense pressure that the pandemic has put on primary care. Increasing demand for appointments alongside GP burnout and declining numbers of GPs were an issue even before the pandemic, and the experiences of the pandemic period have done nothing to ease these issues.^38^ As GPs are central to the diagnosis of cancer,^39^ the smooth running of primary care has implications for the post-pandemic recovery of cancer care. Despite pledges to increase GP workforce by 5000 by 2020 and then 6000 by 2024,^40^^,^^41^ the 2020 target has been missed and 2024 targets are also on track to be missed.^42^ If NHS primary care is to recover, the chronic under-resourcing of services, which is central to many of the reasons that GPs give for leaving the profession, must be tackled directly and with urgency.^42^^–^^44^

In conclusion, the COVID-19 pandemic has had and continues to have a profound effect on primary care and the wider systems that work to detect cancer. Some of the changes will have been a positive step forward in the modernisation of practice, but it is apparent that the net impact of the pandemic on the detection of cancer has been negative and that the full effect on stage, treatment intent, and survival may not be fully understood for some time. There is an urgent need to investigate how changes in diagnostic approaches might affect the patient pathway to diagnosis, especially if changes reduce the GP’s ability to use some of their clinical tools or act as a barrier to help seeking for patients. The pandemic period has not been easy and so it is important that the hard-learned lessons are used to build a more resilient health service.

## Reflexivity statement

The first author is a mixed-methods researcher and has spent 10 years in cancer and primary care research and 12 years in research in total. She has conducted qualitative research with members of the public and with healthcare professionals.
